# Reward processing differences in autism: current understandings and future directions

**DOI:** 10.1093/scan/nsag076

**Published:** 2026-09-15

**Authors:** Kathryn E Unruh, Matthew W Mosconi

**Affiliations:** Life Span Institute and Kansas Center for Autism Research and Training, University of Kansas, Lawrence, KS 66045, United States; Life Span Institute and Kansas Center for Autism Research and Training, University of Kansas, Lawrence, KS 66045, United States; Clinical Child Psychology Program, University of Kansas, Lawrence, KS 66045, United States

**Keywords:** autism, reward, cognition, executive function

## Abstract

Differences in reward processing repeatedly have been implicated in autism. Still, the contexts in which these differences occur (e.g. social and non-social) and the extent to which they relate to autistic traits remain poorly understood. Notably, current frameworks for reward processing have yet to describe the impacts of reward processing differences on broader cognitive domains in autism. Evidence from studies of neurotypical individuals and non-human primates shows that reward has significant impacts on multiple cognitive domains, including executive control processes that have been linked to core social communication and repetitive behavior traits in autism. These findings suggest that examining reward processes in relation to cognitive functions in autism may provide important insights into neural systems that contribute to multiple developmental abilities and psychiatric features common to autism. In this review, we present a new, testable model that describes how interactions between reward and cognitive domains may have additive or synergistic contributions to key clinical traits in autism. This model is framed in context of existing evidence for reward processing differences in autism and their relationship to clinical features, as well as broader implications for expanding existing reward-based frameworks in this population.

Reward processes are the operations that allow an organism to identify and respond to pleasurable stimuli. These operations also are central to goal-directed behavior, supporting the ability to select appropriate responses in the context of changing environmental demands and adapt to individual and situation-specific variations in goal selection. Disruptions of reward processes have been implicated in multiple neurodevelopmental and neuropsychiatric conditions, suggesting that alterations in reward processing systems may have wide ranging impacts on functional behavior and developmental outcomes. Reward processes also have been repeatedly implicated in autism (Chevallier *et al*. 2012, Dichter *et al*. 2012, Kohls *et al*. 2012, 2018, Abrams *et al*. 2013, Dichter 2018), yet the nature of reward processing differences in autism remains poorly understood. Notably, current frameworks for reward processing have yet to describe the impacts of reward processing differences on broader cognitive domains in autism, including executive and inhibitory control processes that previously have been linked to core features of autism. In this review, we first briefly synthesize empirical evidence of reward processing differences in autism and their relationships to autistic traits. Next, we describe an established literature documenting critical interactions between reward and cognitive processes for promoting flexible, goal-directed behavior across development and apply these findings to support a new, testable model of integrated cognitive-reward-based mechanisms of key clinical traits in autism. Finally, we discuss implications of expanding reward frameworks in autism to incorporate developmental context, potential sex differences, and neurodiverse perspectives.

## An integrated cognitive-reward model in autism

An emerging body of literature has demonstrated that reward and cognition are closely and inherently intertwined. Behavioral and neuroimaging evidence demonstrates key differences in both reward processing abilities and multiple cognitive domains in autistic individuals. Despite this, the degree to which reward processing differences in autism may contribute to or interact with cognitive differences has not been well studied, and these domains are rarely assessed within the same individuals. Understanding cognitive-reward interactions in autism is important for several reasons. First, key theories of social-communication features of autism provide strong evidence that differences in both social reward and social cognitive processes contribute to these behaviors, yet few studies have assessed interrelationships between these domains or their cumulative contributions to autistic behaviors. Similarly, select repetitive behaviors in autism have been linked to both reward processing differences (e.g. specialized interests) and neurocognitive deficits that affect behavioral flexibility and inhibitory control (e.g. sameness and compulsive behaviors). While reward processing differences have been theorized to contribute to reduced behavioral flexibility that is characteristic of some repetitive behaviors in autism (Kohls *et al*. 2014), this hypothesis has yet to be explicitly tested. Reward processing differences may contribute to, or exacerbate, previously documented neurocognitive challenges in autistic individuals and may inform mechanistic models for repetitive behaviors and potential intervention approaches.

Second, multiple cognitive difficulties in autism may be exacerbated by differences in reward processing, as is evidenced by the effectiveness of reinforcement-based interventions to promote adaptive behaviors and reduce challenging behaviors for many, but not all, autistic individuals (Cerasuolo *et al*. 2022). Similarly, there may be select contexts in which reward produces paradoxical effects in autistic individuals. For example, the opportunity to engage in a highly preferred activity following completion of a non-preferred task may fail as a motivating strategy if the individual is unable to appropriately allocate attention to the current task because current cognitive resources are “overridden” by focusing on an expected reward. This process may be parallel to the common “choking under pressure” phenomenon (Mobbs *et al*. 2009), in which a substantial incentive can lead to reduced performance. Therefore, a more individualized understanding of distinct neurocognitive profiles and their interactions with reward systems may be useful for personalizing support strategies for autistic individuals.

Third, individual differences in reward processing domains also may contribute to heterogeneity in the expression of autistic features or the experience of co-occurring neuropsychiatric conditions associated with autism. For example, reward-cognitive differences are common in multiple co-occurring conditions that frequently impact autistic individuals, including depression, anxiety, and attention-deficit/hyperactivity disorder (ADHD). Given the overlap between cognitive and reward processing differences across these conditions, select combinations of cognitive and reward differences may be variably present across autistic individuals and helpful in understanding clinical and perhaps neurodevelopmental heterogeneity.

We propose a novel integrative model that evaluates both discrete and synergistic and/or additive contributions of reward and cognitive domains to behavioral features of autism (Fig. 1). By incorporating interactions across domains, we propose that this model may provide greater predictive ability for characterizing psychological and neural correlates of core and associated feature of autism and for understanding individual differences across autistic individuals. Below, we first describe what is currently known about reward processing differences in autism and their relationship to core features. Then, we present evidence for potential disruptions in the interactions between cognitive and reward systems in autism along with testable hypotheses for determining how these interactions may subserve core features of autism or confer risk for commonly co-occurring features. Finally, we introduce additional domains that may be important to consider in advancing this model and other relevant frameworks for understanding reward differences in autism, including developmental impacts, potential sex differences, and input from lived experiences of neurodiverse individuals.

**Figure 1:**
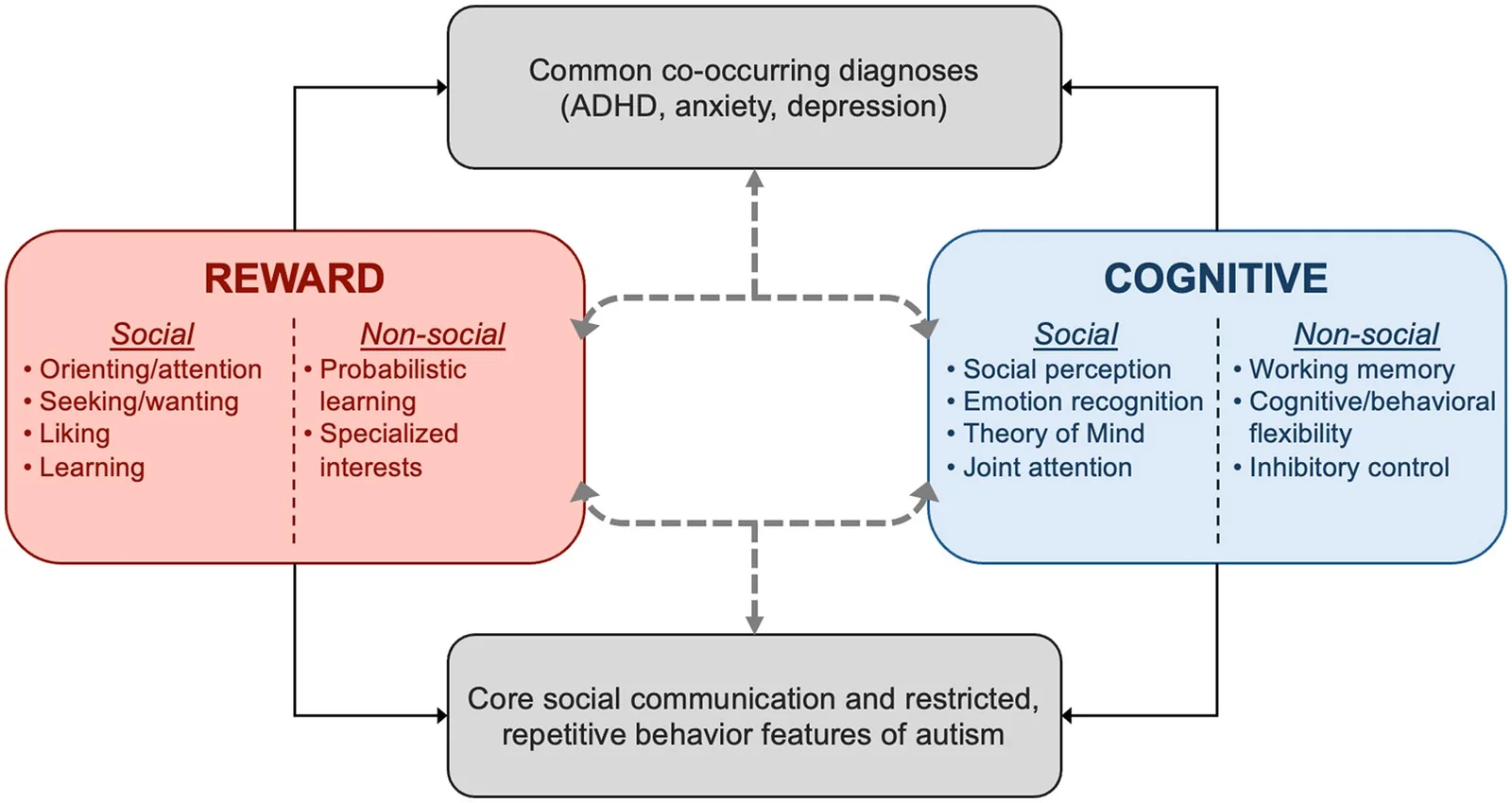
Cognitive and reward contributions to core features of autism. Existing models of cognitive and reward differences in autism consider the isolated influence each of these domains may have on core and co-occurring features of autism (solid black lines). We propose a theoretical integrative model that considers how interactions across cognitive and reward systems may have additive or interacting effects to impact behavior in autism (gray dashed lines). *Note*: Select cognitive and reward processes that previously have been implicated in autism are categorized as social or nonsocial to align with primary findings; however, many of these differences extend across social and nonsocial domains.

## Reward processing and its neural substrates

The term reward can refer to both a positive affective experience as well as the stimuli or events that are likely to elicit these experiences (White 2011). Rewarding events or stimuli may include both external contingencies (e.g. food, money, positive social feedback) and intrinsic processes (e.g. gaining knowledge, mastering a task, completing a project). Studies of external reward contingencies typically evaluate effects of a reward stimulus on behavior, such as through increasing the likelihood of a particular behavioral occurrence (e.g. reinforcement) or through modifying the behavior itself (e.g. reduced reaction time). In contrast, studies of intrinsic reward may examine an individual’s tendency to engage in exploration, spontaneous discovery, and self-directed interest (Lee and Reeve 2017). Quantifying intrinsic reward is difficult, however, contributing to a disproportionate influence of extrinsic reward processes reward-based frameworks.

Reward processing commonly is studied in the context of a three-component model that is dissected into temporally distinct phases with separate underlying neural substrates: (i) anticipating or “wanting” reward, (ii) consuming or “liking” reward, and (iii) reward learning. These processes are subserved by a distributed network that includes striatum, mid-brain dopaminergic regions (ventral tegmental area, VTA), prefrontal cortices, anterior cingulate cortex (ACC), insula, amygdala, thalamus, and sensory and motor cortices (e.g. premotor and occipital cortex). Reward anticipation is associated with engagement of ventral striatal nuclei (nucleus accumbens, NAcc) and VTA, while consumption is more frequently associated with engagement of ventromedial and orbital prefrontal cortices (VMPFC, OFC), though medial (VMPFC and ACC) and insular cortex are frequently reported to show broader engagement across phases (Vassena *et al*. 2014, Pujara *et al*. 2016). Reward learning involves midbrain dopaminergic inputs to striatum and medial prefrontal cortex that signal when actions produce unexpected rewards (Knutson and Cooper 2005), while the value of the reward itself is encoded in OFC (Rolls 2000).

## Reward processing differences and their relationships to core features of autism

### Evidence for social reward differences in autism

Autism is defined by behavioral deficits in core social-communication and restricted, repetitive behavior (RRB) domains. Differences in reward processing and its underlying neural circuitry are frequently linked to autism. Initial theories for the role of reward processing differences in autism were predicated on early emerging differences in social attention. As young as 12 months of age, autistic toddlers show reduced orienting that is selective to naturalistic social stimuli (e.g. faces, name being called), relative to toddlers with non-autistic developmental delays (Dawson *et al*. 1998, 2004, Pierce *et al*. 2023). Autistic toddlers also show a reduced tendency to initiate or respond to bids for joint attention within structured play observations. Developmental cascade models of autism propose that early reductions in social attention result in fewer early developmental learning opportunities that are critical to the maturation of foundational social and communication skills. Both cognition-centric and reward-centric frameworks have been proposed to describe these differences.

The social motivation hypothesis of autism represents a well-studied model which posits that early social attention differences arise from inherent reductions in social reward and that these differences in reward processing underpin a range of social behaviors and social cognitive processes impacted by autism (Chevallier *et al*. 2012). The social motivation hypothesis builds upon the notion that reward is central to behavior and proposes that an inherent motivation for social information begets social behaviors via orbitofrontal–striatal–amygdala circuits that underlie multiple fundamental social and social-cognitive behaviors, including social orienting (prioritizing of social information), social seeking and liking, and social maintaining. This framework positions social cognitive differences observed in autism as a consequence, rather than a cause, of diminished social motivation. This is contrast to cognitive theories of autism that place cognitive or social cognitive (e.g. Theory of Mind) processes as central to autistic behaviors.

Patterns of reduced social attention and initiation offer support for the social motivation hypothesis. For example, a reduced tendency to initiate or respond to bids for joint attention within structured play observations has frequently been observed in autistic toddlers (Mundy *et al*. 1994, Leekam *et al*. 2000). Across childhood and adulthood, passive viewing studies have documented reduced visual attention to socially relevant stimuli (e.g. eyes or faces) relative to background or non-social stimuli (Pelphrey *et al*. 2002, Falck-Ytter 2008, Klin and Jones 2008). Further, reduced attention to social visual and auditory cues early in life is predictive of later autism diagnosis (Pierce *et al*. 2011, 2016, 2023). Preference-based assessments, often quantified using forced-choice viewing arrays, find that autistic individuals show a reduced preference for viewing social images and increased preference for viewing non-social images (e.g. geometric patterns or objects) relative to typically developing peers (Sasson and Touchstone 2014, Pierce *et al*. 2016).

Multiple studies have tested the hypothesis that social information elicits reduced activation of neural reward networks in autistic individuals, relative to neurotypical controls. However, results from these studies have been largely inconsistent, owing to factors such as small sample sizes, variation in comparison conditions (e.g. social vs. monetary reward or social vs. nonsocial reward) and study design (e.g. passive viewing vs. reward-based decision making). To provide consensus across a larger dataset, a meta-analysis from Clements *et al*. (2018) pooled data across task-based functional MRI studies of social and nonsocial reward. Results of the meta-analysis revealed hypo-activations in autistic participants, relative to neurotypical controls, across multiple structures involved in reward processing, including caudate and ACC; however, autistic participants also demonstrated hyperactivations in amygdala and putamen, as well as hypoactivations structures outside of putative reward networks, including hippocampus, lateral occipital cortex, and inferior frontal gyrus and hyperactivations in temporal cortex. During anticipation of social reward (analyses restricted to a subset of studies that probed phasic reward specifically), autistic participants demonstrated a hypoactivation in striatum and prefrontal cortex (anterior PFC and ACC) relative to neurotypical controls. In contrast, autistic participants showed hyperactivations of striatum, limbic nuclei (amygdala and hippocampus) and orbital frontal cortex during phases in which they were “consuming” social rewards (i.e. viewing social images). Findings from this meta-analysis are largely consistent with a subsequent large multi-site study of reward in autistic adolescents and adults, which also reported hypoactivation of ventral striatum during social reward anticipation in autism relative to age-matched neurotypical controls (Baumeister *et al*. 2023). Perhaps the strongest support for reduced encoding of social reward value in autism comes from a study using personalized social and object stimuli, in which authors report selective decreases in VMPFC activation in autistic individuals, relative to neurotypical controls, when viewing images of familiar faces (Kishida *et al*. 2019), while responses to self-selected preferred objects were similar across groups.

Overall, these results point toward reward processing differences in autism that are characterized by reductions in social attention and reduced responses to social stimuli in brain reward circuits for reward anticipation and consumption. However, findings of hyperactivations within other limbic structures (e.g. amygdala and hippocampus) during social reward viewing in autism remain poorly understood and may reflect reduced top-down control of subcortical structures, consistent with affective regulation disruptions (Herrington *et al*. 2016). These findings may provide important insight into distinct psychological experiences (e.g. social avoidance) for at least a subset of autistic individuals, consistent with previously described critiques of the social motivation hypothesis (Jaswal and Akhtar 2018). Future studies that focus on differentiating affective (e.g. anxiety) from reward components and relating patterns of neural activation to individual experience (i.e. social anxiety/avoidance and social interest) are needed to clarify these findings.

### Evidence for non-social reward differences in autism

Multiple studies have suggested restricted or specialized interests evidenced by most autistic individuals may reflect heightened reward in response to non-social objects or interests. Clinically, some autistic individuals show intense interests in select objects or themes (e.g. cars or a specific television show) that may preoccupy attention to a degree that interferes with their ability to maintain reciprocal interactions with their peers (Gabriels *et al*. 2005, Anthony *et al*. 2013) or to engage with contextually relevant information (e.g. school work; Bishop *et al*. 2006, Turner-Brown *et al*. 2011). Importantly, the intensity of these interests also may contribute to severe distress when preferred items are not available or when individuals must be redirected to topics that are less preferred (Klin *et al*. 2007, Sethi *et al*. 2019). These behavioral differences likely involve multiple driving factors including an elevated reward response to preferred objects or topics as well as difficulties with cognitive processes that allow individuals to flexibly shift their attention, thoughts, or behavior. Specialized interests often focus on highly rote or repetitive activities, including gathering factual information, collecting items within a particular category, and being attached to a particular object (Klin *et al*. 2007, Nowell *et al*. 2021). The prominence of these behavioral differences in autism suggests that atypical reward processing may contribute to multiple developmental challenges that are not selectively “social” in nature and that they may interact with neurocognitive functions that have been repeatedly implicated in autism, including attention and executive processes.

Quantitative behavioral studies largely have supported the hypothesis that some autistic individuals show heightened reward responses to specific items or topics relative to non-autistic individuals. During studies in which they are simultaneously presented with social and non-social stimuli, autistic individuals show increased time fixated on non-social objects compared to social objects (Sasson *et al*. 2008, 2011, Unruh *et al*. 2016). Autistic individuals also show a preference for viewing non-social images over receiving monetary rewards whereas non-autistic individuals more frequently prefer monetary rewards (Watson *et al*. 2015). While it is possible that monetary rewards may differ in their perceived value across autistic and non-autistic individuals, collectively these studies suggest that autistic individuals tend to experience greater reward when viewing select non-social objects than their non-autistic peers. However, these studies have failed to identify clinically meaningful associations between the degree to which an individual displays increased interest in (visual attention) or valuation of non-social stimuli and the intensity of repetitive behaviors, including specialized interests. A likely explanation for this lack of association is that while increased engagement with nonsocial stimuli may reflect differences in brain reward systems in autistic individuals, this discrete measure fails to capture the multiple psychological processes that may contribute to repetitive behaviors. This also speaks to the heterogeneity of repetitive behaviors within autism, which are associated with distinct psychological and neural correlates (Lewis and Kim 2009) and may present differently across individuals.

Several studies have tested the hypothesis that autistic individuals show hyper-activations of neural reward circuitry in response to objects that are related to repetitive behavior or restricted interests in autism (Dichter *et al*. 2012, Cascio *et al*. 2014, Kohls *et al*. 2018). Relative to neurotypical controls, increased activations in both cortical (VMPFC, insula) and subcortical (NAcc) regions associated with reward have been reported in autism. These differences extend across studies using personalized stimuli (i.e. using images of items that are specific to an individual’s interests) and non-specific objects commonly associated with special interests in autism (Dichter *et al*. 2012, Cascio *et al*. 2014, Clements *et al*. 2018). Together these findings suggest that select types of nonsocial information are associated with heightened response within brain reward processing networks, as evidenced by hyperactivations of regions associated with both anticipatory (NAcc) and consummatory (VMPFC) reward. Increased response within insula in autistic individuals is reported to be associated with interference of specialized interests in daily life, consistent with the role of insula in reward-based decision making, including allocation of attention to rewarding stimuli (Rothkirch *et al*. 2014), suggesting that some object-related information may be more motivationally “salient” to autistic individuals. Together these data are consistent with behavioral and self-report data from autistic individuals indicating that engaging in specialized interests provides a significant degree of satisfaction and enjoyment (Patten Koenig and Hough Williams 2017, Grove *et al*. 2018).

### Evidence for reward learning differences in autism

Reward learning is the process by which an organism acquires or strengthens associations between behaviors and rewards or, in the context of unexpected non-receipt of reward, is biased toward seeking new associations, with the overall purpose of orienting attention to maximize future rewards. Reward learning is facilitated through phasic midbrain dopaminergic activity, which encodes reward prediction errors (RPE) that signal about the expected and received value of rewards throughout striatum and cortex (Schultz *et al*. 1997, Glimcher 2011). RPE signaling occurs when a mismatch is detected between expected and received outcomes; positive prediction errors occur when a reward is received or better than expected, while negative prediction errors signal a worse than expected outcome. In this latter condition, RPE signals may instead bias motor planning systems toward acquisition of new associations via dopaminergic inputs to medial cortical structures, including ACC, thus promoting behaviors that are flexible and goal-directed (Holroyd and Coles 2002).

Reduced rates of reward learning have been reported in autistic children and adults, particularly when acquiring social, relative to monetary, reward contingencies (Scott-Van Zeeland *et al*. 2010, Lin *et al*. 2012, Choi *et al*. 2015, Kinard *et al*. 2020). However, impairments in feedback-based learning using nonsocial contingencies also have been reported (Solomon *et al*. 2011, 2015). Relative to neurotypical peers, autistic individuals demonstrate atypical activations across striatal-medial prefrontal circuits during reward learning tasks that are explicitly social in nature (Scott-Van Zeeland *et al*. 2010, Choi *et al*. 2015, Balsters *et al*. 2017, Kinard *et al*. 2020), though the directionality of these differences is not consistent across samples. In contrast, nonsocial reward learning is associated with hyperactivation in insula and frontal pole in autism, relative to neurotypical peers (Mosner *et al*. 2019). Striatal differences during reward learning in autism suggest a disruption in the generation of RPEs, consistent with behavioral evidence showing that autistic individuals do not effectively use feedback-related information during tests of behavioral flexibility (D’Cruz *et al*. 2013). During reward-based decision making, insula response is associated with ambiguity and uncertainty (Simmons *et al*. 2008), and findings of increased insula activation during RPE in autistic individuals may indicate that learning and prediction error processes underlie clinical features of intolerance of uncertainty in autism.

### Key limitations of reward studies in autism

#### Lack of ecological validity

The study of reward is inherently limited by the feasibility of including ecologically valid stimuli, particularly for MRI study designs. Social interactions critically involve unpredictable information and rapid assessment of changing contextual cues, which may contribute to anxiety or psychological discomfort with naturalistic social situations for some autistic individuals. These features are difficult to capture during experimental evaluations; viewing of social images is a largely predictable, low-stimulation experience, and this disparity in representations of reward stimuli may contribute to a lack of consistent findings for reward differences in autism and incomplete understanding of the nature of social reward differences.

To address this, a recent study by Chiappini *et al*. (2024) tested neural reward response to tangible social (interpersonal touch) and nonsocial (flavored drinks) stimuli in autism. Relative to neurotypical peers, autistic participants demonstrated multiple hyper-activations that were specific to sensory-related cortical areas (parietal, occipital, and superior frontal), rather than in reward-associated circuits. One possible interpretation of this data is that differences in social interaction and engagement observed in autism may be attributed to mechanisms such as sensory over-responsiveness or difficulty with rapid information processing. Though speculative, such findings call into question whether social motivation differences observed in autism can be attributed to inherently “social” nature of social interactions and experiences, or whether social contexts are more often enriched with sensory inputs or involve multiple competing cognitive demands. These considerations are of critical importance in redefining previous misconceptions about autistic individuals’ desire for social relationships and social understanding.

Studies of nonsocial reward also may be limited in their interpretation based on a lack of standardization in the reward value of monetary reward across autistic and non-autistic individuals. For example, non-autistic individuals with a greater degree of autonomy and financial independence may value money differently than individuals with reduced opportunities to interact with financial reward. Studies using “fixed economies” based on points systems that are not directly tied to specific financial values have been useful in mitigating monetary value differences in non-autistic adolescents (e.g. Geier and Luna 2012) but have not been adopted in studies of reward in autism.

## Interactions of cognitive and reward processes

Adaptive, flexible behavior, supported by executive functions, involves assessing the value of actions and integrating these assessments to adjust behavior to changing environmental demands. These value assessments are a means by which reward interacts with higher-order cognitive processes, such as attention and executive functions. For example, reward may interact with attentional processes when an individual is tired during a long road trip and more likely to identify signs for a coffee shop amidst many road signs. Reward also influences decision making according to individual goals, such as when deciding whether to adjust a thermostat during warm weather and increase short-term comfort, or prioritize reduced financial costs in the future by limiting immediate changes in temperature. Cognitive-reward interactions have been well-documented in lab-based tasks that use external reward contingencies (e.g. monetary reward) to improve performance on cognitive tasks in a dose–dependent manner (Engelmann *et al*. 2009, Krebs *et al*. 2010, 2012, Padmala and Pessoa 2010, Engelmann and Pessoa 2014). Although the mechanisms of cognitive-reward interactions are still being elucidated, findings indicate that offering reward contingent upon task performance is associated with changes in subcortical and midbrain circuits involved in reward processing (e.g. VTA dopaminergic regions) and “domain-specific” changes in cortical activations, such as within frontal-parietal attentional control networks and occipital-temporal visual cortical networks during rewarded spatial localization tasks (e.g. Engelmann *et al*. 2009). These data suggest that external reward contingencies serve to improve behavioral performance by interacting with both top-down (attentional) and bottom-up (sensory) processes to guide behavior. Behavior is similarly guided by internal reward cues. For example, during a target detection task, internal reward cues such as hunger, are associated with increased activations in frontal and parietal cortices to food-related stimuli, and these changes in activation are largely absent when individuals are sated, suggesting that an individual’s internal state can modulate spatial attention systems similarly to external reward (Mohanty *et al*. 2008). These data suggest that experimental manipulations of reward (e.g. monetary incentives) are useful for simulating the influence of various motivational states on behavior.

### Neural mechanisms of cognitive and reward interactions

The integration of cognitive and reward processes relies on direct and indirect communication between task-relevant cognitive and reward networks (Botvinick and Braver 2015). Striatal dopamine appears to lie at the interface of direct interactions between cognitive and reward networks (Haber *et al*. 2000, Aarts *et al*. 2014). Functional subdivisions of striatum are defined based on their cortical inputs in a ventromedial to dorsolateral gradient, corresponding to reward, cognitive, and motor circuits. Striatum also receives dense projections from midbrain dopaminergic structures. Parallel striatal–cortical and striatal–midbrain circuits do not function discretely; rather tracer studies have demonstrated spiraling striatal–midbrain–striatal and striatal–cortical–striatal loops in rodents and non-human primates that allow information to flow across reward, cognitive, and motor structures (Haber *et al*. 2000, Haber 2003, Ikemoto 2007; Fig. 2). Initial preclinical findings demonstrated that dopaminergic signaling to ventral striatum modulates activity in dorsal striatum (Nauta *et al*. 1978), by way of VTA-ventral striatal projections that form a central component of the mesolimbic reward pathway (Haber *et al*. 2000). These findings suggest that that dopaminergic innervation of ventral striatum facilitates a feed-forward flow of information by which reward impacts cognitive and motor control processes via spiraling striatal–midbrain–striatal and striatal–cortical–striatal loops (Haber 2016). In this way, striatal integration of cognitive and reward signals can occur at the level of midbrain–striatal dopaminergic loops or at the level of frontal–striatal connections. Ventral striatum also receives dense projections from amygdala and hippocampus, which contribute to the ability to integrate information about reward memory, context, and affective state to promote both approach and avoidance behaviors (Haber and Knutson 2010, Levita *et al*. 2012).

**Figure 2:**
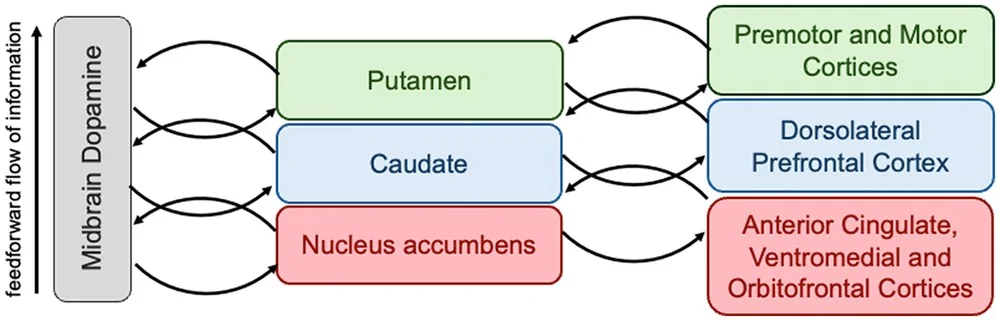
Cognitive–reward interactions occur at the level of striatum. Striatal loops between midbrain dopaminergic regions and cortex support interactions across reward (red), cognitive (blue), and motor (green) control networks. Dopaminergic signals facilitate a feedforward flow of information in a ventral to dorsal direction, providing a mechanism by which reward signals exert influence over cognitive domains.

Midbrain dopaminergic signaling also facilitates indirect communication across cognitive and motor networks. Under conditions of reward, diffuse dopaminergic modulation is associated with increased cross-network coupling of task-relevant networks (e.g. Kinnison *et al*. 2012) that promote improvements in cognitive processes such as attention, working memory, and executive functions (Kawagoe *et al*. 1998, Lauwereyns *et al*. 2002, Takikawa *et al*. 2002, Jazbec *et al*. 2005, Krawczyk *et al*. 2007, Kennerley and Wallis 2009, Jimura *et al*. 2010, Infanti *et al*. 2015, Manohar *et al*. 2015, Wallis *et al*. 2015, Gong *et al*. 2017, Hallquist *et al*. 2018, Brissenden *et al*. 2023). These improvements are consistent with neuromodulatory effects of dopamine that include the ability to alter neuronal excitability (Henze *et al*. 2000, Nicola *et al*. 2000) and enhance signal to noise ratios (Vander Weele *et al*. 2018). Therefore, reward-mediated impacts on cognition such as increased perceptual sensitivity or enhanced attention appear to be facilitated by dopaminergic signals. Consistent with this hypothesis, improved tactile discrimination under conditions of reward is associated with increased neural activity in primary somatosensory cortex (Pantoja *et al*. 2007, Pleger *et al*. 2008) that is mediated by dopaminergic inputs as evidenced by their selective enhancement or attenuation following administration of dopamine agonists or antagonists, respectively (Pleger *et al*. 2009). Additionally, individual differences in striatal dopamine synthesis, receptor density, and baseline dopamine levels have been linked to differences in cognitive phenotypes (Bilder *et al*. 2004, Colzato *et al*. 2010) and degree to which reward impacts cognitive control, including when reward produces detrimental effects (Aarts *et al*. 2010). Further, the effects of dopamine on cognitive processes appear to follow a U-shaped curve, in which both insufficient and excessive dopaminergic signaling are associated with poorer task performance (Cools and D’Esposito 2011). Still, mechanisms underlying reward-related reductions in performance are not yet clear and warrant further study.

Select brain structures also may serve as hubs of cognitive-reward integration based on receiving both reward and task-relevant inputs (Barbas and Pandya 1989, Cavada *et al*. 2000, Botvinick and Braver 2015). ACC broadly subserves response monitoring processes, including computing the difference between an expected and actual outcome of a reward to inform the need to change response strategies. One theory proposes that ACC may link motivation to cognition by computing the expected value of exerting cognitive resources, relative to the potential outcome and the degree of effort required (Shenhav *et al*. 2013). Lateral PFC also has been proposed as a hub of integration that facilitates the modulation of cognitive processes according to reward context. This notion is supported by evidence that dopaminergic inputs to lateral PFC promote the use of proactive control strategies, which prime the cognitive networks for task demands according to task contexts, in contrast to more reactive strategies that are stimulus driven (Braver and Cohen 2000, O’Reilly and Frank 2006). Considering the role of lateral PFC in maintaining accurate representation of task goals (Barbey *et al*. 2013), reward-based dopaminergic inputs therefore may be involved in maximizing proactive control strategies in the context of high reward conditions.

### Implications of cognitive and reward interactions in neuropsychiatry

Disruptions in the interactions between cognitive and reward processes may have multiple behavioral implications within neurodevelopmental and neuropsychiatric disorders. For example, in patient with schizophrenia, an inability to accurately represent expected reward values (e.g. differentiate between small and large rewards) is proposed to selectively impair reinforcement learning, while other types of learning remain spared (Robison *et al*. 2020). Similarly, in ADHD and other impulsive disorders, an increased preference to choose small, immediate rewards over larger, delayed rewards, which may reflect a reduced ability to integrate inhibitory control and reward processes (Ma *et al*. 2016). The extent to which disruptions in reward processes may interact with cognitive differences in autistic individuals has not been studied. Below we describe what is known about cognitive-reward interactions in neurotypical individuals across two key processes: working memory and inhibitory control, and discuss how known differences in each of these processes may be considered in the context of reward differences in autism.

### Evidence supporting an integrative cognitive-reward model in autism

#### Structural and functional differences in striatum

Alterations in striatal morphometry, including volumetric increases and divergent developmental trajectories, are among the most well-documented structural brain differences in autistic individuals and show multiple linkages to clinical behaviors (Sears *et al*. 1999, Hollander *et al*. 2005, Rojas *et al*. 2006, Langen *et al*. 2007, Sato *et al*. 2014, Evans *et al*. 2024). For example, multiple independent samples have demonstrated that larger striatal volume is associated with more severe clinically rated RRBs (Sears *et al*. 1999, Hollander *et al*. 2005, Rojas *et al*. 2006, Langen *et al*. 2009, 2014, Estes *et al*. 2011, Qiu *et al*. 2016). Altered functional connectivity of striatum also is common and is reported in both autistic children and adults (Di Martino *et al*. 2011, Delmonte *et al*. 2013, Abbott *et al*. 2018, Balsters *et al*. 2018, Akkermans *et al*. 2019, Bartolotti *et al*. 2020), including differences in developmental trajectories (Padmanabhan *et al*. 2013, Ma *et al*. 2022). Specific to the current model, increased NAcc-PFC intrinsic (task-free) connectivity has been reported in two large samples of autistic children and adults (*N* = 219 and *N* = 119, respectively; Nakamura *et al*. 2023, Li *et al*. 2025), while another study reported decreased functional connectivity and reduced white matter density in these tracts based on a smaller sample of autistic individuals (*N* = 24; Supekar *et al*. 2018). These data implicate alterations in both bottom-up, reward-driven and top-down control circuitry that is central to feed-forward integration of striatal reward signaling into cognitive and motor control domains.

Emerging evidence also suggests that integration across striatal circuits is affected in autism and associated with core clinical features. Using task-free functional connectivity measures that were converted to z-scores, Abbott *et al*. (2018) identified that the ratio connectivity in frontal–striatal reward, relative to frontal-striatal cognitive circuits differed in autistic youth relative to age-matched peers. Further, greater connectivity within reward-related circuits relative to cognitive-related circuits was associated with increased rates of ritualistic, compulsive, and sameness behaviors in autistic participants. These data suggest that studies characterizing both discrete and integrated function across striatal circuits may be needed to advance a mechanistic understanding of select repetitive behaviors in autism.

#### Interactions across reward, working memory, and learning processes

Multiple studies have documented effects of reward processing on working memory abilities. Working memory supports the retention and manipulation of information over brief periods and involves maintenance and retrieval processes. Working memory supports social-communication abilities in multiple ways, including storage of temporally ordered linguistic information, maintenance of social goals during an interaction (e.g. turn-taking or conversational prompts), the ability to incorporate multiple pieces of social information (e.g. affective state of the social partner, context, and content of the conversation), and capacity for organizing and planning social responses (Barkley 1997, Barrett *et al*. 2004, McQuade *et al*. 2013). In non-autistic individuals, working memory abilities are predictive of social competencies (McQuade *et al*. 2013) and social problems (Kofler *et al*. 2011).

Storage of memory during retention periods is supported by sensory cortices according to the content of the memory stimulus (e.g. lateral temporal cortex supporting semantic working memory; Binder *et al*. 2009). Dorsolateral PFC (DLPFC), is critical to the maintenance of working memory information and supporting the retention of information despite distractors or interference (Barbey *et al*. 2013). Reward signals are associated with enhancement of key perceptual information that may be important for working memory processes (e.g. spatial location, color, or other select details) and in supporting sustained activity in prefrontal circuits during working memory maintenance. For example, in nonhuman primate studies of delayed visual recall tasks, the maintenance of task-relevant information (e.g. target location) is supported by increased firing rate of location-specific neurons in DLPFC, which show sustained activity throughout delay periods. Leon and Shadlen (1999) found that when select visual target locations were associated with large rewards, corresponding neurons in DLPFC showed increased firing rates. These data suggest that reward may selectively enhance perceptual features to aid in working memory encoding and retrieval processes. This hypothesis is consistent with findings from human studies showing that extrinsic reward is associated with increased activations in PFC during working memory encoding periods, as well as in perceptually relevant cortices such as the fusiform face area of temporal cortex during facial recognition memory tasks (Krawczyk *et al*. 2007). Individuals with greater self-reported intrinsic motivation also show differences in neural dynamics during working memory tasks relative to those who are less motivated, characterized by an increase in activity in lateral prefrontal cortex during both encoding and maintenance periods (Jimura *et al*. 2010).

Many studies have documented reduced working memory abilities in autistic individuals relative to non-autistic peers (see Kercood *et al*. 2014), including deficits in retaining both visual-spatial and phonological information (Williams *et al*. 2005, Steele *et al*. 2007, Cui *et al*. 2010, Macizo *et al*. 2016, Habib *et al*. 2019). Working memory deficits are reported both in autistic individuals with and without intellectual disability (Landa and Goldberg 2005), suggesting that these challenges may be central to autism rather than a function of co-occurring cognitive impairments. Despite the centrality of working memory abilities to social cognition (Meyer and Lieberman 2012), few studies have documented associations between working memory abilities and core features of autism (Landa and Goldberg 2005, Lopez *et al*. 2005, Sachse *et al*. 2013). These data suggest that relationships between working memory differences in autism and features of the disorder may be mediated by additional factors.

##### Existing evidence and implications for future research

Solomon *et al*. (2015) tested the hypothesis that flexible, goal-directed learning challenges in autism may reflect an increased reliance on reinforcement learning processes and inability to engage working memory systems to guide learning under contexts of unpredictable feedback. Using a probabilistic learning paradigm in which participants must learn stimulus-response associations that are presented with varying rates of accurate feedback (probabilistic), this study reported that autistic individuals were slower to learn stimulus-response pairs relative to neurotypical controls. Neurotypical controls also demonstrated increased recruitment of superior and anterior prefrontal cortices during probabilistic learning, consistent with a strategy of engaging PFC to hold reward-based information online to determine the accuracy of feedback across multiple trials (Collins *et al*. 2017). In contrast, autistic individuals showed greater recruitment of ACC and OFC relative to controls, implicating increased reliance on trial-to-trial reinforcement learning processes (McDannald *et al*. 2014). This type of learning is slower and incremental, relying on trial-to-trial feedback to establish new patterns. As learning becomes more complex, such as in the case of goal-directed behaviors, there is greater need to recruit frontal and parietal circuits involved in the maintenance and manipulation of information to accommodate more complex, less predictable feedback (Collins *et al*. 2017). In the context of social-communication challenges that many autistic individuals experience, these data provide preliminary evidence that autistic individuals exhibit a reduced ability to hold reward-based information in working memory to guide more complex behaviors, and instead rely on prediction-error based learning processes that limit the ability to carry out dynamic, flexible social interactions.

Neuroimaging studies of working memory in autism find that memory deficits are associated with reduced task-related activation in DLPFC and posterior parietal cortex (Luna *et al*. 2002, Vogan *et al*. 2018) and reduced frontal–parietal connectivity (Solomon *et al*. 2009). During working memory maintenance, dopaminergic reward signals contribute to selective enhancements in DLPFC neurons that code for key perceptual features, such as location (Kodama *et al*. 2014). Reduced DLPFC activations during working memory in autism, therefore, may reflect reductions in dopaminergic reward signals that perpetuate a reduced ability to consolidate key representations needed to maintain or manipulate online information. For example, in the context of social interactions, working memory and social cognitive differences may be exacerbated by reductions in social motivation, resulting in poor social experiences that further promote a reduced ability to experience social reward (Fig. 3). This hypothesis is supported by previous findings that social motivation is predictive of social skills in autistic children, even when controlling for broader cognitive deficits (Itskovich *et al*. 2021). Future studies examining social motivation and social cognitive abilities are needed to test hypothesis. Pharmacological challenge studies also may be useful for testing the effects of dopaminergic reward systems on working memory and broader cognitive skills in autism based on the broad use of stimulants in autistic individuals. These studies are predicated on data in ADHD showing that stimulant use, which increases dopamine in striatum and PFC, is associated with increased working memory abilities (Wong and Stevens 2012) and select improvements in social cognition (Alkalay and Dan 2022).

**Figure 3:**
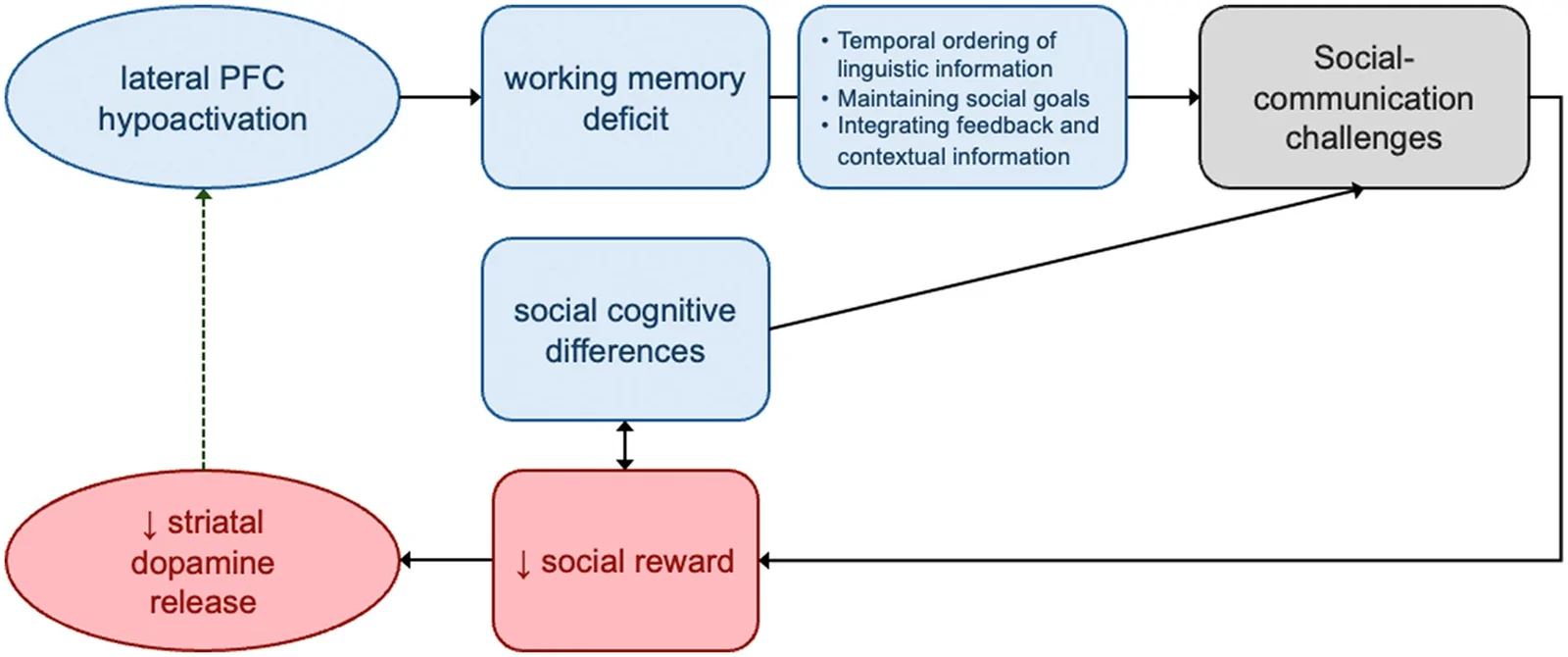
Hypothetical model for cognitive–reward interactions in the context of social-communication behaviors in autism. Working memory supports multiple behaviors involved in complex social interactions. During social interactions, working memory deficits and social-cognitive differences in autism may contribute to social-communication challenges that result in non-rewarding social interactions. Poor quality social interactions may perpetuate or contribute to a reduced experience of social reward for autistic individuals, which in turn may contribute to existing cognitive deficits (dotted line).

#### Interactions across reward and inhibitory control processes

Inhibitory control describes the processes by which an individual suppresses habitual, prepotent, or task-irrelevant behaviors. Inhibition may be proactive, involving goal-directed planning processes (e.g. driving slower through a busy intersection in anticipation of having to stop), or reactive, such as in response to a stimulus (e.g. stopping when the light turns red). Studies of non-autistic individuals have shown that extrinsic reward can support inhibitory control abilities, and that the degree to which reward processes modulate inhibitory control abilities varies widely across individuals and development (Geier *et al*. 2010, Padmanabhan *et al*. 2011, Hallquist *et al*. 2018). Notably, proactive control abilities appear to be more sensitive to dopaminergic modulation based on studies showing that goal-directed and goal-maintenance processes that support proactive behavior are mediated via dopaminergic inputs to prefrontal cortex (Braver and Cohen 2000, Braver *et al*. 2014). In contrast, reactive control processes that are largely stimulus-driven may be less sensitive to dopaminergic modulation (Braver *et al*. 2014).

Inhibitory control deficits have been well-documented in autism and are associated with multiple core and co-occurring clinical traits. Existing models of RRBs in autism propose that select repetitive behaviors arise from a reduced ability to suppress preferred, but contextually inappropriate behaviors (Lopez *et al*. 2005, South *et al*. 2007). Numerous studies have documented support for this model showing that reduced inhibitory control abilities are associated with a greater tendency to exhibit select clinical RRBs including ritualistic, insistence on sameness, and compulsive-type behaviors, or “higher-order” RRBs, but not stereotypic motor behaviors (Mosconi *et al*. 2009, Mostert-Kerckhoffs *et al*. 2015, Faja and Nelson Darling 2019). Despite these findings, associations between inhibitory control deficits and clinical RRBs are moderate in their effect size, predicting between 20% and 50% of variance across studies, indicating that inhibitory control differences in autism contribute to RRBs, but that separate mechanisms also are involved. Aside from studies examining reward response to specialized interests, no studies have explicitly tested whether reward processing differences may contribute to “higher-order” RRBs in autism. However, based on numerous studies linking alterations in striatal morphometry and development to RRBs in autism (Sears *et al*. 1999, Hollander *et al*. 2005, Rojas *et al*. 2006, Langen *et al*. 2009, 2014, Estes *et al*. 2011, Qiu *et al*. 2016), it has been posited that reward processing differences may be important targets. Specifically, Kohls *et al*. (2014) proposed that RRBs such as compulsions and insistence on sameness behaviors are highly rewarding to autistic individuals and that the continued selection of these behaviors over others may lead to a reduced ability to select novel or alternate behaviors. This model suggests that existing inhibitory control deficits may be exacerbated by reward processing differences in autism that contribute to an over-valuation of prepotent or reflexive behaviors. Studies that evaluate an individual’s own reward valuation of specific habits and routines are needed to test this hypothesis.

##### Existing evidence and implications for future research

Two existing studies provide preliminary evidence to support the hypothesis that reward processing differences may have interactive effects on inhibitory control deficits in autism. Saniee *et al*. (2025) tested whether reward impacted proactive control processes that support selective attention toward task-relevant (and away from task-irrelevant) stimuli during a test of cognitive flexibility in autistic children. The task required children to shift attention across various task features (e.g. shape and color) to select correct responses across trials. Under no-reward conditions, autistic youth performed more poorly than age-matched neurotypical controls; however, providing rewards contingent upon correct performance significantly increased task performance in both participant groups. This study provides preliminary evidence that engaging neural reward systems may mitigate select cognitive issues in autism, including those that underlie inhibitory control deficits. Pankert *et al*. (2014) tested the effects of social reward contingencies on inhibitory control in autistic youth and age-matched neurotypical peers. This study reported reward-contingent improvements in inhibitory control across all participants, with autistic individuals showing a greater degree of benefit relative to neurotypical peers, though these effects were variable across participants and some demonstrated paradoxical effects (poorer performance). Mothers of autistic participants who demonstrated worse inhibitory control during rewarded trials endorsed using more reward-based strategies in the home environment. This unexpected finding may suggest a disconnect in cognitive-reward system integration in some autistic individuals, in which a high degree of motivation produced more invigorated behavior that led to increased error rates, at the expense of effective inhibitory control (Manohar *et al*. 2015). Studies that further examine individual differences in the extent to which reward improves, or exacerbates, behavioral deficits may be important for personalizing intervention strategies for autistic individuals.

Neuroimaging studies in autism also support the hypothesis that reward processing differences may partially underlie inhibitory control deficits. ACC exerts top-down control over PFC during inhibitory control (Johnston *et al*. 2007). During antisaccade paradigms in which the participant is asked to inhibit a reflexive saccade toward a peripherally appearing distractor stimulus, autistic adults and adolescents exhibit reduced functional connectivity between ACC and frontal eye fields (FEF), in addition to reduced pre-trial activations of FEF (Agam *et al*. 2010, Padmanabhan *et al*. 2015). In the context of preclinical studies demonstrating that pre-trial FEF activity is associated with proactive control strategies supporting successful inhibition of a reflexive saccade (prosaccade) and subsequent volitional generation of an antisaccade (Curtis and D’Esposito 2003, Van der Stigchel *et al*. 2012), these findings indicate coordination of top-down control mechanisms necessary for goal-directed behavior, such as those driven by intrinsic reward processes, are reduced in autism and may underlie inhibitory control deficits. ACC also is involved in response monitoring and error detection processes needed for integrating reward-based feedback into ongoing actions (Polli *et al*. 2005). Studies of autistic children and adults have demonstrated reduced sensitivity of ACC in response to errors, relative to age-matched controls during multiple tasks involving inhibitory control (Thakkar *et al*. 2008, Sokhadze *et al*. 2010, Goldberg *et al*. 2011, Santesso *et al*. 2011). Although inhibitory control paradigms, such as antisaccade tasks, are not explicitly rewarded, real-time sensory-perceptual feedback may modulate performance. Based on this premise, these data suggest that inhibitory control deficits in autism may involve a reduced ability to integrate feedback signals, whether intrinsic or extrinsic, to modify ongoing behavior.

Together these data suggest that autistic individuals may demonstrate a reduced ability to effectively use reward, or other feedback-related information, to support cognitive control processes. Studies that examine the effects of reward contingencies on volitional (e.g. antisaccade) and reflexive (e.g. prosaccade) behaviors will be useful for clarifying whether existing inhibitory control deficits in autism are exacerbated by a hyper-response to reward (e.g. faster saccades that are less accurate when expecting reward), or reflect hypo-responsivity to reward feedback (e.g. saccades are neither faster nor more accurate with reward contingencies). Measuring associations between these lab-based behaviors and clinical measures of core and associated features of autism will be useful for determining discrete vs. synergistic contributions of inhibitory control and reward processes to clinical behaviors.

#### Parallel findings in conditions that co-occur with autism

##### Major depressive disorder

Depressive disorders are common among autistic individuals (lifetime rates estimated at 14%–48%; Hudson *et al*. 2019) and associated with deficits across both cognitive and reward domains, including difficulty with executive functions (e.g. diminished ability to engage in goal-directed behavior; Degl’Innocenti *et al*. 1998, Snyder 2013) and anhedonia (i.e. diminished interest or pleasure in activities). Rumination, a symptom of depression that involves repetitive thought patterns about negative feelings, distress, and their causes and consequences, has been linked to both reward and inhibitory control dysfunction. In the context of an interactive cognitive-reward model, rumination may reflect a failure to inhibit negative self-referential thought that is exacerbated by differences in the ability to process reward-based feedback. This proposed mechanism parallels our previously hypothesized model for RRBs in autism that involves a reduced ability to inhibit engaging with sameness-type behaviors that are highly rewarding.

Consistent with this hypothesis, a small number of studies have examined ruminative tendencies in autistic individuals and their associations with features of autism and co-occurring depression diagnoses. Autistic individuals report higher levels of rumination than non-autistic controls that are positively associated with autism trait severity (Patel *et al*. 2017), including sameness-type RRBs (Gotham *et al*. 2014, Ibrahim *et al*. 2019). Additionally, antidepressants have been shown to reduce ruminative thought patterns in patients with depression (Segerberg *et al*. 2024) and, in select cases, reduce insistence on sameness behaviors in autism (Najjar *et al*. 2015). These data suggest a shared mechanism by which potential disruptions in cognitive and reward processes may confer shared risk for both autistic and depressive features. Longitudinal studies that track insistence on sameness symptoms and the development of depressive symptomology are needed for testing these hypotheses. Pharmacological studies also are needed to test whether the treatment of depressive features also serves to mitigate RRBs in autism.

##### Attention-deficit/hyperactivity disorder

ADHD is common among autistic individuals (26%–40%; Canals *et al*. 2024, Rong *et al*. 2021, Yerys *et al*. 2025) and disruptions in inhibitory control and reward processing each have been proposed as candidate mechanisms of core inattention, impulsivity, and hyperactivity symptoms (Barkley 1997, Nigg *et al*. 2005, Sonuga-Barke 2005). Core symptoms of ADHD have been linked to reward circuitry dysfunction (striatal hypo-activation) across multiple independent samples (Scheres *et al*. 2007, Ströhle *et al*. 2008, Hoogman *et al*. 2011, Carmona *et al*. 2012), suggesting that reduced sensitivity to reward may contribute to inhibitory control deficits in this population. Consistent with this hypothesis, individuals with ADHD demonstrate multiple behavioral improvements when correct performance is associated with reward contingencies, including faster and less variable reaction times and improved response inhibition (e.g. Michel *et al*. 2005, Huang-Pollock *et al*. 2007, Liddle *et al*. 2011, Rosch *et al*. 2016). Treatment studies also provide preliminary evidence that cognitive features of ADHD are mitigated by targeting brain reward networks, as opposed to those subserving cognitive control. For example, methylphenidate treatment in ADHD, which affects striatal and prefrontal cortical dopaminergic signaling, is associated with multiple changes in brain reward networks including restoring “neurotypical-like” connectivity patterns across reward and attention networks during tests of attentional and cognitive control (Rubia *et al*. 2009, Mizuno *et al*. 2023).

The neurocognitive profile of autistic individuals with co-occurring ADHD is poorly understood, and many autistic individuals report high rates of ADHD symptoms even in the absence of clinical diagnosis. Autistic individuals and those with ADHD share many cognitive impairments. The presence of ADHD may be associated with a greater degree of impairment in working memory or proactive control domains (Sinzig *et al*. 2008, Yerys *et al*. 2009), while reactive control may be more selectively associated with features of ADHD (Van Hulst *et al*. 2018). Autistic individuals with high rates of ADHD symptoms also striatal hypoactivation during anticipation of monetary reward, similar to non-autistic individuals with ADHD (van Hulst *et al*. 2017). These data suggest that select combinations of reward and cognitive differences (e.g. reactive control deficits and hyposensitivity to reward) may represent overlapping mechanisms in autism are predisposed toward the development of ADHD features. Studies that characterize reward and neurocognitive phenotypes within autistic individuals with and without elevated features of ADHD are needed to test this hypothesis.

##### Anxiety

More than one in three autistic children and adolescents have been diagnosed with an anxiety disorder (van Steensel *et al*. 2011), though up to 80% of autistic individuals may experience clinically elevated features of anxiety (Nadeau *et al*. 2011). Impaired emotional regulation is linked to anxiety in both autistic and non-autistic youth (Mennin *et al*. 2003, Cai *et al*. 2018, Conner *et al*. 2020), a process that is associated with disruptions in cognitive control (Ochsner *et al*. 2012). Behaviorally, emotional regulation disruptions involve a tendency to ascribe threat to neutral stimuli and avoidance of potentially threatening situations (Walkup *et al*. 2021). Emotion regulation processes are subserved by a distributed network involving amygdala and its connections with medial and lateral prefrontal cortex (Etkin 2010). Amygdala hyper-reactivity to emotional stimuli and disruptions in top-down PFC control of amygdala each are frequently reported in anxiety, suggesting increased threat detection that is not well-regulated by cognitive control systems (Monk *et al*. 2008, Gold *et al*. 2015, Xie *et al*. 2021). Nucleus accumbens is involved in active avoidance of threats (Levita *et al*. 2012, Wenzel *et al*. 2015) and receives direct projections from amygdala (Haber and Knutson 2010). Threat signaling processes, much like reward processes, also involve projections from midbrain dopaminergic regions to striatum (Salamone 1994, Menegas *et al*. 2018). Therefore, anxiety appears to involve disruptions between cognitive control and reward systems in which hyper-responsivity of amygdala in the context of a perceived threat, coupled with reduced top-down control of PFC, may promote increased avoidance behavior via amygdala inputs to NAcc, which has hierarchical control over cognitive and motor striatal circuitry (Haber *et al*. 2000).

Though treatment of anxiety commonly involves cognitive-based interventions (e.g. cognitive behavioral therapy) and pharmacotherapies are associated with changes in attentional and cognitive control networks (Maslowsky *et al*. 2010, Burkhouse *et al*. 2018, Strawn and Levine 2020), differences in reward sensitivity and in the ability to use reward to guide future behavior each are associated with differences in treatment response for anxiety in both non-autistic and autistic youth with anxiety (Sequeira *et al*. 2021, Hollocks *et al*. 2023, Berg *et al*. 2025). These findings are particularly salient in the context of data showing that autistic individuals with anxiety show reduced benefit of both psychotherapy and pharmacotherapy interventions (Bowers *et al*. 2015, Patel *et al*. 2020), suggesting that existing differences in reward processing may both predispose some individuals to anxious tendencies and moderate treatment outcomes. Ongoing efforts to improve personalized intervention approaches for autistic youth (e.g., Beversdorf and MISSOURI AUTISM SUMMIT CONSORTIUM 2016) may benefit from considering individual differences in both cognitive and reward profiles in relation to treatment outcomes.

### Further opportunities to expand reward-based frameworks in autism

#### Relationships between reward processing development and early cognitive abilities

Studies of older children and adults have informed the majority of our understanding of reward processing differences in autism. This is due, in part, to a limited understanding of the emergence of reward processing in neurotypical development. Reward processing differences in autism may be considered using a developmental framework proposed by Clements *et al*. (2023) that outlines six core components that promote the emergence of reward processing and the ability to reliably assess anticipation, response, and learning. According to this model, infants must develop the abilities to form associations and understand contingencies, discriminate between stimuli, demonstrate preference or understanding of value, exert effort, anticipate, and respond. Longitudinal studies of these components that track their development in autism and association with later validated measures of social and nonsocial reward have not yet been conducted. However, existing studies do provide evidence for disruptions across multiple early component processes in young children with autism or infants who later receive an autism diagnosis, including reductions in the abilities to discriminate between sensory stimuli (Guiraud *et al*. 2011, Piccardi *et al*. 2021, Espenhahn *et al*. 2023), make anticipatory eye movements (Landry and Bryson 2004), and generalize associations across stimuli (Northrup *et al*. 2017). Also well-documented in autism are early motor disruptions, which have been linked to the later development of multiple core features of autism, and are hypothesized to contribute to a reduced ability to learn new contingencies via environmental exploration or exert effort to obtain rewards (West 2019, Lim *et al*. 2021). The 6-component model provides an important framework by which early cognitive differences in autism may predispose disruptions in reward processing. Studies that map these components across early development and test their associations with early emerging aspects of social motivation and cognitive development are needed to test hypotheses about the origins of reward processing differences in autism and their potential cognitive precursors.

#### Exploration of sex differences

The majority of research on reward processing in autism is informed by data from male participants, consistent with higher rates of autism diagnoses in males compared to females. However, relative to autistic males, autistic females self-report higher levels of social interest and friendship quality (Sedgewick *et al*. 2016) and demonstrate a greater degree of insight into social relationships (Mattern *et al*. 2023), suggesting that social reward processing differences in autism may vary across sexes. Despite this, only two studies have explicitly examined sex differences in neural reward circuitry in autism.

When viewing social images, autistic females show increased activation in NAcc, relative to autistic males, and in insula and lateral prefrontal reward regions, relative to non-autistic females (Lawrence *et al*. 2020). Findings of increased activation across separate frontal cortical circuits associated with processing salient stimuli suggest that autistic females may prioritize seeking out and obtaining social feedback to a greater extent than same-sex non-autistic peers, and that models of social reward processing in autism may vary across females and males. Resting state functional connectivity differences in reward networks also are reported in autistic females, relative to autistic males and neurotypical females. Hernandez *et al*. (2020) reported that relative to autistic males, autistic females show greater connectivity of NAcc with other structures involved in the reward network, as well as with prefrontal cortical structures involved in cognitive control, showing connectivity patterns that are overall more similar to neurotypical males than autistic males. In autistic females, increased connectivity between NAcc and lateral prefrontal structures involved in cognitive control also is associated with better social-cognitive ability, suggesting that the ability to integrate across cognitive and reward systems may support social cognitive abilities in these individuals. Together these studies provide strong preliminary data to indicate that reward processing differences are not unitary across autistic individuals and that enriching female participants to examine sex effects is a critical focus of future research.

#### Consideration of neurodiverse perspectives

Current frameworks for social reward processing in autism are informed by neurotypical understandings social contexts and inherently designed to test a deficit-based model that may not accurately reflect the lived experience of autistic individuals. For example, assertions that autistic individuals are less socially motivated than their neurotypical or non-autistic developmentally delayed peers are largely predicated on findings of reduced frequency of social behavior or attention among autistic people. Recently, critiques of the social motivation hypothesis have suggested that while autistic individuals may “appear” less socially interested, behavioral indices of social interest have been defined from a neurotypical perspective (Jaswal and Akhtar 2018). Considering this alternate perspective, behaviors such as lack of response to name and differences in how eye contact is modulated during social interaction may not necessarily represent a lack of social interest or motivation, but instead may reflect separate factors such as anxiety, difficulty responding to unpredictability, or preference to focus attention on one source of information (e.g. auditory inputs) rather than integrating across multiple modalities (e.g. visually tracking eye movements or facial gestures). Although autistic adolescents and adults tend to report lower levels of social interest relative to typically developing peers, many autistic individuals also report high levels of social interest. Some autistic individuals also report high levels of loneliness, which is a strong predictor of depression in autistic youth (Deckers *et al*. 2014, 2017, Han *et al*. 2019). Further, autistic individuals who self-report greater social reward and show higher levels of autism symptomology report increased loneliness relative to autistic peers who have fewer autism symptoms (Han *et al*. 2019). Reports of loneliness in autistic individuals are inconsistent with an interpretation of reduced social motivation and suggest an unmet desire for social connection. Yet, existing cognitive or social cognitive differences in autism may result in low quality social interactions. These interpretations suggest that cognitive supports and interventions may provide a great degree of benefit for some autistic individuals, relative to interventions that selectively target social reward pathways (e.g. intranasal oxytocin).

#### Intrinsic motivation differences

In contrast to external reward contingencies frequently used in laboratory reward paradigms (smiling faces, monetary reward, viewing images), intrinsic rewards reflect internally generated experiences, such as those that arise from feelings of pride and accomplishment for completing a task, mastering a skill, experiencing a sense of belonging. Autistic individuals may have a reduced opportunity to experience intrinsic reward across multiple contexts due to factors such as social exclusion and reduced opportunity to seek employment in careers that cater to their individual strengths and interests (Jones *et al*. 2022, Davies *et al*. 2024). Intrinsic reward is important not only for increasing task engagement, but also for promoting autonomy, feelings of competency, and other factors that are central to self-determination (Di Domenico and Ryan 2017). Multiple studies have reported lower self-determination in autistic youth relative to non-autistic peers and those with other developmental disabilities (Wehmeyer and Shogren 2008). Reduced self-determination in autism may arise from multiple factors, and there is currently no literature explicitly linking reward processing differences to perceptions of self-determination in autistic individuals. However, conceptual frameworks for self-determination intervention strategies illustrate the way in which integrating supports for cognitive challenges in autism, such as reductions in inhibitory control or cognitive flexibility, may promote self-determination abilities (Shogren *et al*. 2021), and theoretically, increase intrinsic reward. Such frameworks demonstrate clinical applications for understanding cognitive-reward interactions in autism, wherein cognitive gains may impact an individual’s experience of intrinsic motivation, and represent an important opportunity for promoting strengths-based perspectives, in favor of deficit-based models.

## Conclusions

An expanding body of work has demonstrated that reward processes are closely and inherently involved in modifying cognitive processes in support of flexible and goal-directed behavior. These findings suggest that underlying differences in either reward processing, cognitive ability, or a combination thereof, may have additive or synergistic effects on behavior in autism. Current reward- and cognition-based frameworks in autism provide multiple opportunities for considering how differences in each of these domains may affect the other, yet few studies have explicitly tested this hypothesis. Existing studies of reward in autism provide evidence that autistic individuals experience both social and nonsocial reward differently than non-autistic peers, yet the nature of those differences remains poorly understood. We propose that studying cognitive differences in the context of existing reward differences in autism may advance a more mechanistic understanding of autistic features. For example, social cognitive and working memory differences each may contribute to difficulties during social interactions for some autistic individuals and result in social experiences that are non-rewarding; in turn, reduced social reward may further exacerbate cognitive difficulties in these contexts and perpetuate these challenges. Similarly, heightened response to select rewards, coupled with a reduced ability to inhibit preferred behaviors, may give rise to inflexible patterns of behavior that cannot adapt to changing environmental demands. Studies that systematically evaluate this novel framework are needed to define key psychological and neural correlates of core and associated features of autism, develop more personalized intervention approaches, and understand individual differences that may confer risk for select psychopathologies.

## Data Availability

No new data were generated or analyzed as part of this study.
